# Case report: Uterine perforation caused by migration of intrauterine devices

**DOI:** 10.3389/fmed.2024.1455207

**Published:** 2024-09-05

**Authors:** Qi Li, Desheng Qi, Tingting Bi, Xinyi Guo, Huan Chen

**Affiliations:** ^1^Department of Gynaecology, Zhuzhou Central Hospital, Zhuzhou, China; ^2^Department of Traditional Chinese Medicine, Women and Children Healthcare Hospital of Zhuzhou, Zhuzhou, China; ^3^Department of Radioimaging, Zhuzhou Central Hospital, Zhuzhou, China

**Keywords:** migration of intrauterine devices, uterine perforation, laparoscopy, hysteroscopy, three-dimensional computed tomography

## Abstract

Intrauterine devices (IUDs) are often considered a form of contraception by women of reproductive age because of their reversible, effective, safe, and convenient nature. However, its complications include bleeding, infection, displacement, and uterine perforation. As most patients do not exhibit any obvious symptoms, they ignore their complications and are unaware of the necessity of regular evaluation. Therefore, they are unable to implement timely interventions for the complications that can result in serious consequences. Although, three-dimensional (3D) ultrasound has demonstrated greater sensitivity in detecting subtle IUD malposition issues, particularly with side-arm embedment. Computed tomography (CT) scanning followed by multi-planar reformatting, maximum intensity projection, and volume rendering can precisely and intuitively display the morphology and location of the IUD, accurately exhibit the anatomical relationship between the IUD and the pelvis, and allow for a more accurate assessment of the degree of perforation and presence and absence of bowel perforation, thereby enabling us to select a more suitable surgical procedure with less damage to the patient. In this study, we reported an asymptomatic case of uterine perforation of the IUD into the serosal layer of the bladder, which developed 6 years post-IUD placement. A preoperative 3D reconstruction was made using the CT images of the IUD; then, the IUD was successfully removed with the assistance of a hysteroscope and laparoscope.

## Introduction

Effective contraception can minimize the risk of unintended pregnancy, particularly for women who have additional health risks associated with unintended pregnancy. Intrauterine device (IUD) is considered to be a highly effective and reversible method of contraception (long-acting reversible contraceptives) that is suitable for most women, highly effective, affordable, and requires minimum patient compliance (1). Despite being infrequent, perforation constitutes a significant complication associated with IUD insertion, with an estimated incidence of 0.8–2.2 per 1,000 insertions (2, 3). Ultrasound is the preferred diagnostic modality in cases where partial embedding of the IUD is suspected. However, when the IUD extends near or beyond the uterine serosa, radiologists can more confidently diagnose embedding (4). Standard radiologic methods are generally effective in localizing most IUDs. Recently, the diagnostic accuracy of ectopic IUDs has been significantly improved by introducing multislice computed tomography (CT) and its associated post-processing techniques. Additionally, this technological advancement has simplified clinical procedures for the surgical intervention or removal of ectopic IUDs (5). This case describes the use of CT three-dimensional (3D) reconstruction to locate the patient’s ectopic IUD accurately. This enabled us to select a more appropriate surgical procedure with less damage to the patient.

## Case description

The patient, a 33-year-old woman, was admitted to the hospital because a routine physical examination revealed a displaced IUD after 6 years of IUD placement. She was asymptomatic and did not experience any discomfort. Her past medical history included two cesarean sections in 2011 and 2015. In 2016, a year after the second cesarean section, a MYCu IUD was placed for contraception about 1 month after discontinuing breastfeeding. One month after MYCu IUD placement, a color Doppler ultrasonography of the uterus and adnexa revealed a normal IUD position. Following that, she had no routine gynecological examinations. In August 2022, during a routine physical examination, a color Doppler ultrasound of the uterus and bilateral adnexa exhibited a displaced IUD with embeddedness into the uterus muscularis propria, which resulted in hospitalization.

## Diagnostic assessment

**Figure 1:**
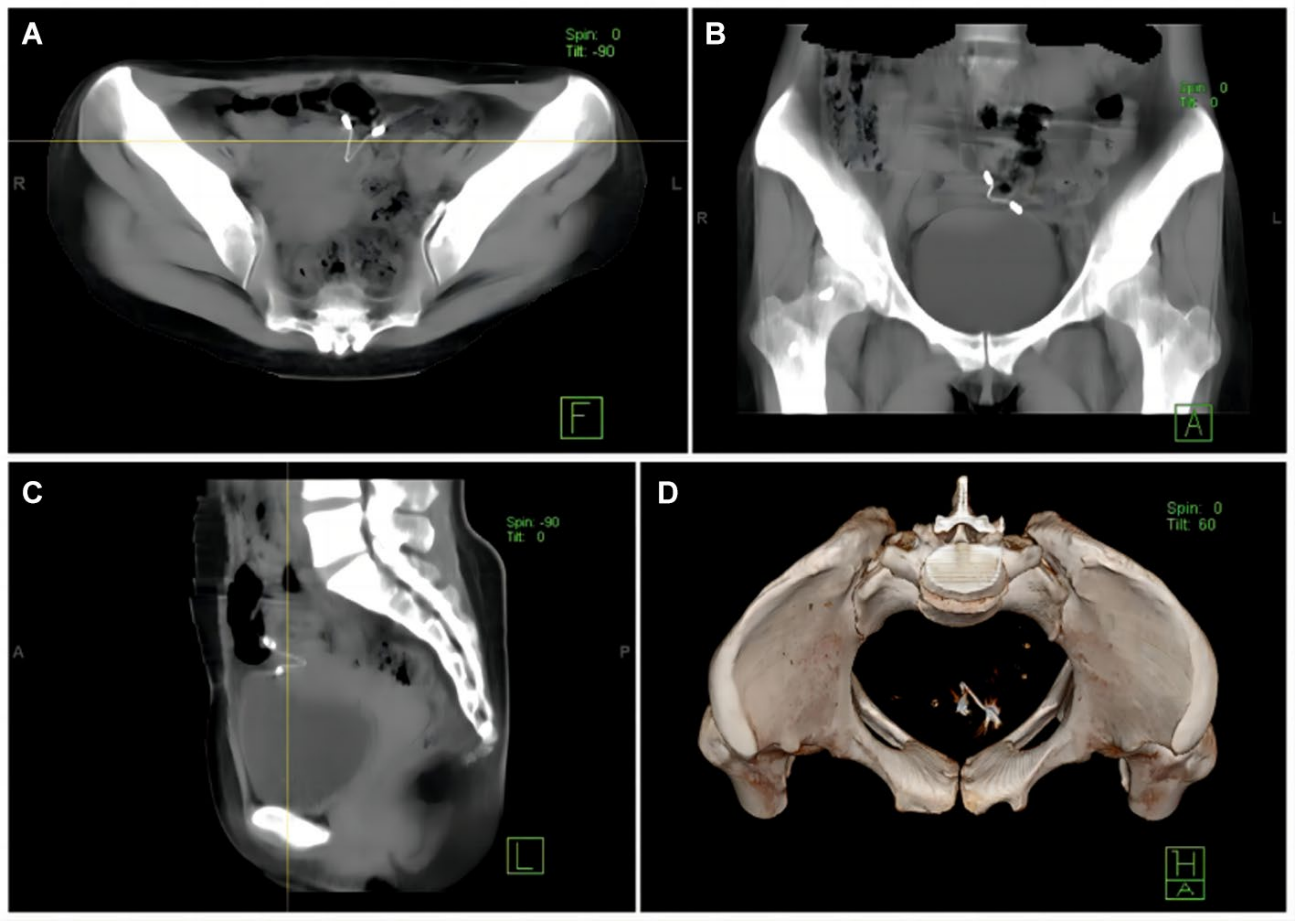
**(A)** A CT image illustrates the poorly demarcated displaced IUD from the adjacent bowel and bladder. **(B)** A CT image indicates that the displaced IUD is closely located in the serosal layer of the bladder. **(C)** The IUD has migrated outside the uterus, and part of the stent is located in the uterine cavity. **(D)** CT 3D reconstruction exhibits that the IUD is located to the left of the pelvis.

A gynecological examination after admission revealed no abnormality. Doppler ultrasound of the uterus and bilateral adnexa exhibited a strong echo of the IUD in the upper left part of the uterine cavity extending to the intermuscular wall and partially projecting out of the uterine silhouette, close to the bladder wall, with a thickness of the endometrium of about 6 mm. Pelvic spiral CT (model: SOMATOM Emotion 16) exhibited a V-shaped hyperdense shadow in the left wall of the uterus surrounded by radiologic artifacts. The outer end breached the uterine wall, which was poorly demarcated from the adjacent bowel and bladder (Figure 1). A routine stool and urine analysis revealed weakly positive fecal and urine occult blood. Preoperative diagnosis indicated dislocation and insertion of IUD and scarred uterus.

**Figure 2:**
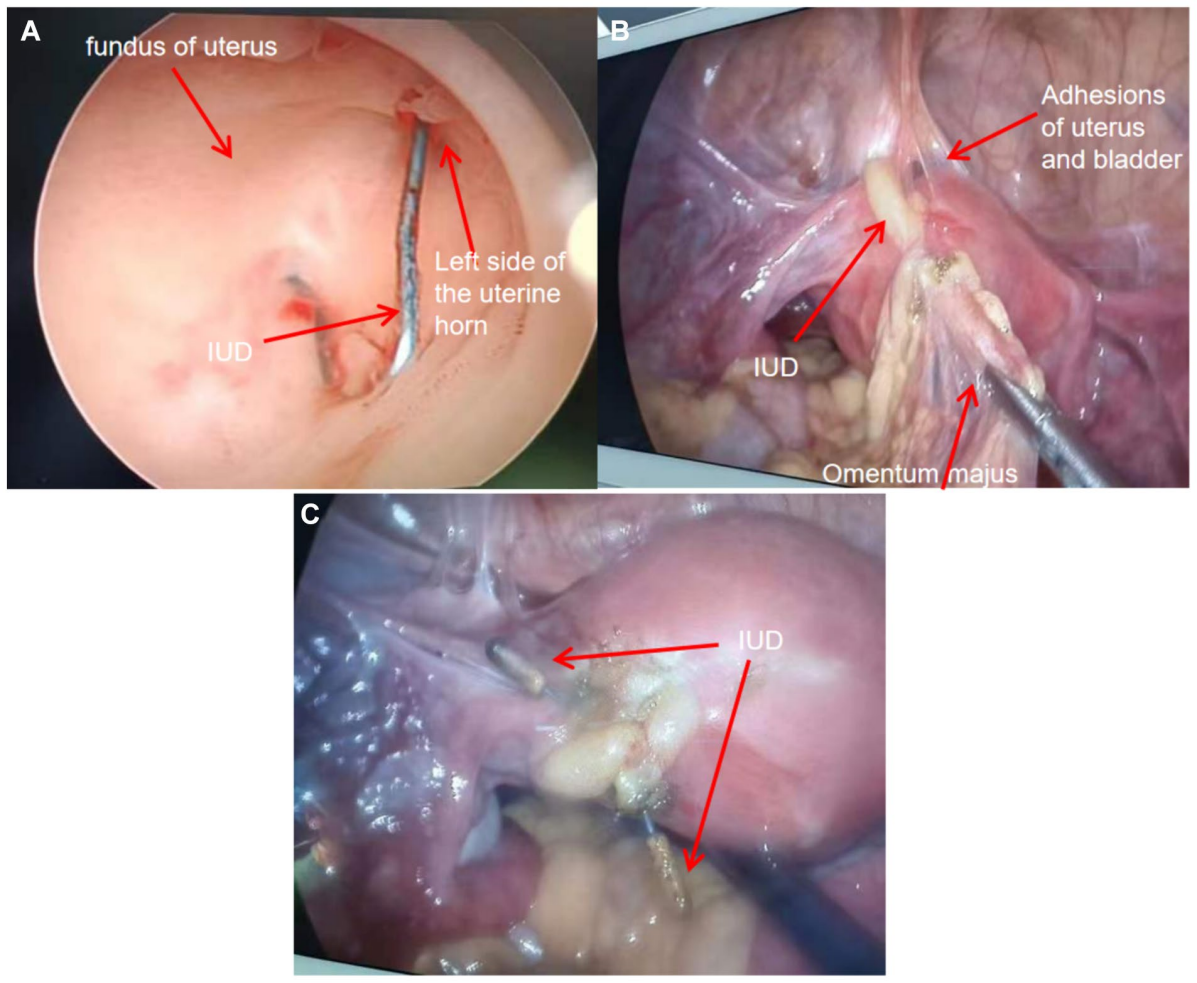
**(A)** Intraoperative hysteroscopic view of the left uterine horn indicating the part of the “MYCu” stent with both arms penetrating the myometrium. **(B)** Intraoperative separation of some adhesions revealing uterine bladder adhesions and the copper particles at the end of one arm of the IUD stent encapsulated in the greater omentum. **(C)** Exposure of both arms of the IUD after separation of adhesions. Both arms completely penetrated the left uterine horn.

The hysteroscopy and laparoscopy procedures were conducted on the third day following the diagnosis. Intraoperative hysteroscopy revealed an “MYCu” partial stent in the left uterine horn, and its two arms penetrated the myometrium (Figure 2A). Laparoscopy indicated that the left uterine horn near the round ligament adhered to the bladder and the uterine wall densely, and the left uterine fundus contained the greater omentum and intestines. One copper arm of the MYCu IUD was observed to have penetrated the serosal layer of the bladder through the vesicouterine peritoneal reflection, while the other copper arm was encapsulated in the greater omentum (Figure 2B). After separating some of the adhesions, the copper arm was liberated. Both arms of the MYCu IUD were completely exposed in the abdominal cavity (Figure 2C). The MYCu IUD was completely removed through hysteroscopy under laparoscopic observation. The intraoperative bladder injection (Melan solution) test indicated no leakage from the bladder. Additionally, there was no damage to the bowel. The operation was successful, and routine *cefmetazole* anti-infective treatment was administered postoperatively. A urinary catheter was left in place for 2 weeks following the operation. The catheter was removed after 2 weeks, and the patient had no urinary fistula. Color Doppler ultrasound of bladder residual urine was normal on follow-up.

## Discussion

In contemporary practice, IUD placement is one of the most prevalent contraceptive methods used by women globally. However, the utilization of IUDs is associated with multiple complications, including infections, uterine bleeding, pelvic abscesses, and uterine perforation (6).

Vaginal bleeding is the most prevalent complication associated with IUDs, attributed primarily to the compression exerted by the IUD on the local endometrial tissue. This compression can cause minor damage to the uterine mucosa, resulting in bleeding and subsequently hindering the repair of the detached endometrium. Consequently, patients may experience increased menstrual bleeding and incomplete shedding of the endometrial lining (7). The incidence of lower abdominal pain induced by IUDs ranks second only to that of vaginal bleeding. The primary mechanism underlying this pain is uterine contraction in response to the foreign body. Furthermore, the risk of lower abdominal pain can be increased by the significant pressure on the endometrium that may result from uterine infections that may arise during the placement process or from an improper matching of the IUD model (8, 9). IUD infection is characterized as a localized inflammatory response that develops postoperatively in the absence of preoperative inflammation within the reproductive system, caused by inadequate adherence to intraoperative aseptic protocols (10). Uterine perforation is defined as the penetration of the device into the plasma membrane layer of the uterus. The IUD implantation into the muscularis propria is relatively uncommon, with occurrence rates of 0.1–0.9% (11). Uterine perforation represents a severe complication of IUD incarceration and extra-uterine ectopic pregnancy, particularly following a cesarean section. This condition is attributed to the thinning of the myometrial layer and the disruption of uterine scar continuity post-cesarean section. In conjunction with surgical adhesions and uterine stretching and deformation, the heightened risk of uterine perforation is exacerbated by the defective healing of the uterine scar, alterations in the myofibrillar structure, and the resulting weakness and reduced elasticity of the myometrial wall (12). Some studies have found that a very small number of IUD perforations are asymptomatic and are often detected during a routine physical examination (13, 14). These asymptomatic perforations are more likely to have serious consequences if left untreated.

The mechanism of IUD uterine perforation, preventive measures, and treatment modalities require additional investigation. The primary risk factors for IUD implantation or displacement are breastfeeding, insertion within 6 months of delivery, uterine abnormalities, history of previous pelvic surgery, post-menopause, and inflammatory conditions. Intraoperative or early perforation or displacement during IUD insertion depends on the surgeon’s experience (14). The uterus is softer during lactation and postpartum, the myometrial tissue is fragile and prone to injury, and the uterus contracts during lactation. Consequently, even a minor injury can result in IUD perforation, particularly in patients with a history of multiple cesarean sections. According to a study of the biomechanics associated with IUD placement, metal IUDs are more likely to perforate the uterus than plastic IUDs with the same force (15). Additionally, it is one of the intrinsic reasons for uterine perforation of IUDs.

In all patients with complications, including perforation and displacement, aggressive surgical removal of the IUD is recommended to avoid serious consequences (16, 17). Routine surgical procedures include hysteroscopy, laparoscopy, and in extremely rare cases of displacement to the bladder, cystoscopy or even cesarean section (11). In cases of perforated IUDs during pregnancy, laparoscopic removal under local anesthesia is a feasible and safe alternative to consider (18). Accordingly, in the case of IUD perforation of the uterus displaced to the pelvic or abdominal cavity, comprehensive preoperative examination and evaluation are essential for both the patient and the surgeon. Frequently used methods to examine IUDs include color Doppler ultrasound, abdominal radiography, and CT. Particularly when side-arm embedment is involved, 3D ultrasound has demonstrated greater sensitivity in identifying subtle malposition issues in IUDs (19). It is becoming a standard practice in the routine evaluation of IUDs (20). In conventional radiography, minimal radiation is emitted, and radiopaque IUDs are readily identifiable if they have not been expelled (20). A CT scan can not only help radiologists in identifying a correctly placed IUD and assessing its integrity, but it can also evaluate for any potential complications, including malposition, embedding, perforation, adhesions, obstruction, abscess, and involvement of other structures within the abdomen following IUD perforation (4). CT scanning followed by multi-planar reformatting (MPR), maximum intensity projection (MIP), and volume rendering (VR) can precisely and intuitively display the morphology and location of the IUD. Besides, it can accurately indicate the anatomical relationship between the IUD and the pelvis and allow for a clearer judgment of the degree of perforation and the presence and absence of bowel perforation. Some patients can be selected for CT 3D reconstruction technology irrespective of the occurrence of perforation (21, 22). The use of magnetic resonance (MR) imaging for evaluating an IUD is uncommon; however, imaging can be useful for locating the IUD and determining its relationship with the uterus (20). A systematic evaluation has been conducted on the impact of women with copper-containing IUDs in clinical MR and CT scanners. The results indicate that MR examinations are safe for women with copper IUDs under the conditions that were tested. The image quality of CT is more affected than that of MR imaging and needs to be carefully considered during the diagnostic process (23).

In conclusion, it is recommended that an adequate evaluation should be performed before IUD placement and that the appropriate time be determined. Moreover, post-operative health education is crucial for women with IUD placement. Regular checkups are recommended for women with IUD placement. Patients should undergo IUD displacement or perforation if no IUD is detected in the uterine cavity on color doppler ultrasonography following IUD placement or if there is a pregnancy with IUD or symptoms such as abdominal distension, pain, urinary frequency, urgency, or dysuria. The gynecologist should conduct a comprehensive preoperative evaluation in patients with complete uterine perforation before making an individualized decision regarding the procedure.

## Data Availability

The original contributions presented in the study are included in the article/supplementary material, further inquiries can be directed to the corresponding author.
